# Analysis of gene co-expression networks of phosphate starvation and aluminium toxicity responses in *Populus* spp.

**DOI:** 10.1371/journal.pone.0223217

**Published:** 2019-10-10

**Authors:** Thiago Bergamo Cardoso, Renan Terassi Pinto, Luciano Vilela Paiva

**Affiliations:** Central Laboratory of Molecular Biology, Department of Chemistry, Federal University of Lavras, Lavras, Brazil; University of Toronto, CANADA

## Abstract

The adaptation of crops to acid soils is needed for the maintenance of food security in a sustainable way, as decreasing fertilizers use and mechanical interventions in the soil would favor the reduction of agricultural practices’ environmental impact. Phosphate deficiency and the presence of reactive aluminum affect vital processes to the plant in this soil, mostly water and nutrient absorption. From this, the understanding of the molecular response to these stresses can foster strategies for genetic improvement, so the aim was to broadly analyze the transcriptional variations in *Poupulus* spp. in response to these abiotic stresses, as a plant model for woody crops. A co-expression network was constructed among 3,180 genes differentially expressed in aluminum-stressed plants with 34,988 connections. Of this total, 344 genes presented two-fold transcriptional variation and the group of genes associated with those regulated after 246 hours of stress had higher number of connections per gene, with some already characterized genes related to this stress as main hubs. Another co-expression network was made up of 8,380 connections between 550 genes regulated by aluminum stress and phosphate deficiency, in which 380 genes had similar profile in both stresses and only eight with transcriptional variation higher than 20%. All the transcriptomic data are presented here with functional enrichment and homology comparisons with already characterized genes in another species that are related to the explored stresses, in order to provide a broad analysis of the co-opted responses for both the stresses as well as some specificity. This approach improves our understanding regarding the plants adaptation to acid soils and may contribute to strategies of crop genetic improvement for this condition that is widely present in regions of high agricultural activity.

## Introduction

Agriculture in acid soil regions is hampered by several factors, mostly related to the absorption of essential nutrients and water by the plants, especially due to Al^3+^ ions in soils with pH below 5.5 that causes toxicity and inhibits root growth. Besides the toxic effect, free aluminium ions in acid soils forms complexes with phosphate, resulting in low availability of this nutrient for the crops.[1]. Approximately 30% of the arable land of the planet is composed of this type of soil [2], which predominates in tropical and subtropical regions, precisely the regions with the highest agricultural activity [3].

The free aluminium resulting from the low pH, in the form of Al^3+^, can interact with several extra- and intracellular components of plant cells, impairing signalling pathways and transport of molecules, leading to changes in cell walls and plasma membranes, with particularly high morphological impact on the roots [4], whose growth is affected, thereby reducing the absorption of water and nutrients. In addition, phosphate starvation in acid soils severely affects plant growth and development, because phosphorus is an essential molecule in most metabolic processes [5]; thus, these soils require regular applications of fertilizers.

The application of phosphate fertilizers has increased in recent years due to food production, which causes concern due to the risks of soil exhaustion as well as the unequal global distribution of phosphate reserves [5]. In this context, strategies focused on the adaptation of plants to this cultivation condition may be of interest for maintaining food security in a sustainable manner. To that end, understanding the molecular mechanisms associated with tolerance to acid soils may be useful for genetic improvement focused at increasing the yield of crops grown in this environment.

It is reasonable that some molecular responses to aluminium toxicity and phosphate starvation stresses may be co-opted, as these stresses co-exist in the same environment and the most directly affected organ is the root in both cases[6,7]. Some findings pointing to co-opted responses have already been reported, like the exudation of organic acids by root tips, that chelates Al^3+^ ions and at the same time increase phosphate availability [8], activation of the same transcriptional regulator triggered by both stresses [9] and possibilities of hormone signalling crosstalk [10].

There are already reports in the literature of applications of molecular knowledge about adaptation to acid soils in commercially relevant crops [11–13] Here we focus on studying the broad transcriptomic response to aluminium and phosphate starvation stresses in *Populus ssp*. as a model plant for woody crops [14,15], due to its genetic, genomic and transcriptomic data availability [16,17], established procedures for tissue culture techniques and genetic transformation [18], including the already reported applicability of novel gene editing techniques [19–21], which will facilitate further proofs-of concept for the hypothesis explored in this work. Altogether, the approach is aimed to improve the adaptation of major woody crops cultivated in acid soils, like coffee, eucalyptus, pinus and others.

In view of the above, this work highlights the transcriptional responses to aluminum toxicity and phosphate starvation in *Populus* spp. by the use of public microarray data and points to connections between the responses to the two stresses through gene co-expression networks. This approach is justified by the importance of understanding the relationship between the responses of these two major coexisting stresses in acidic soils and the lack of studies that address this relationship, as well as the responses to each stress in a specific way, in broad transcriptomic data.

A co-expression network was constructed with differentially expressed genes in response to aluminum toxicity and phosphate starvation, with 550 genes connected by 8,380 connections with ρ≥0.8. With this, we identified groups of genes specifically regulated in each stress that exhibited distinct characteristics. Of the total, 380 genes were identified with the same regulatory profile for both stresses, but only eight had transcriptional variation higher than 20%, four of them similar to genes already characterized in the literature as related to phosphate starvation [22–24]. These findings and the other specific observations explored in this work are useful for specific molecular studies that may support breeding programs in the adaptation of plants to acid soils.

## Results and discussion

Currently available tools for analysis of transcriptomic data (e.g., *microarray* and *RNA-seq*) together with knowledge on the genome and physiology of model plants enable a broader understanding of a given stress impact on plant metabolism, as already demonstrated with the gathering of data from four transcriptome-wide analysis experiments in *A*. *thaliana*, for an approach that revealed 95 genes commonly activated by phosphate deficiency [13].

With a similar aim, microarray data from *Populus* roots exposed to 500 μM Al^+3^ for 6, 54 and 246 hours were used [25]. These data were analysed and 5359 probes with differential expression in at least one of the libraries were identified, among which 3272 were linked to genes already annotated in the *P*. *trichocarpa* genome (S1 Table). Due to the large number of genes found, the two-fold up or down variation between aluminium-stressed and control condition plants was used as the criterion to select only the genes with the highest response to the presence of Al. With this, only 344 genes were considered as regulated by the Al, including either positive or negative regulation (S1 Table). This regulation is influenced by the duration of stress, and we found some genes that were exclusively regulated (either down or upregulated) at specific time points (Table 1).

**Table 1:** General description of the genes.

	6h of stress	54h of stress	246h of stress
**Upregulated genes**	62 (44 exclusive)	52 (10 exclusive)	45 (8 exclusive)
**Downregulated genes.**	250 (148 exclusive)	31 (6 exclusive)	48 (5 exclusive)

General description of the number of genes with differential regulation as a function of aluminium stress at different exposure times.

A higher number of genes were regulated at 6 h of stress, mainly negatively, which may have relation to the stress perception and the initial plant response, as well as the severe root growth arrest noticed in the plants at this time point of the experiment [25]. Within the group of downregulated genes, there was an eight-fold reduction in the number of regulated genes between the interval of 6 and 54 h, and the number increased again in the interval of 54 to 246 h. This early regulation of aluminium stress response has been observed in other species via genome-wide transcriptome analyses (RNA-seq), such as *Fagopyrum esculentum* [26], *Medicago sativa* [27] and *Urochloa decumbensis* [28].

**Fig 1:**
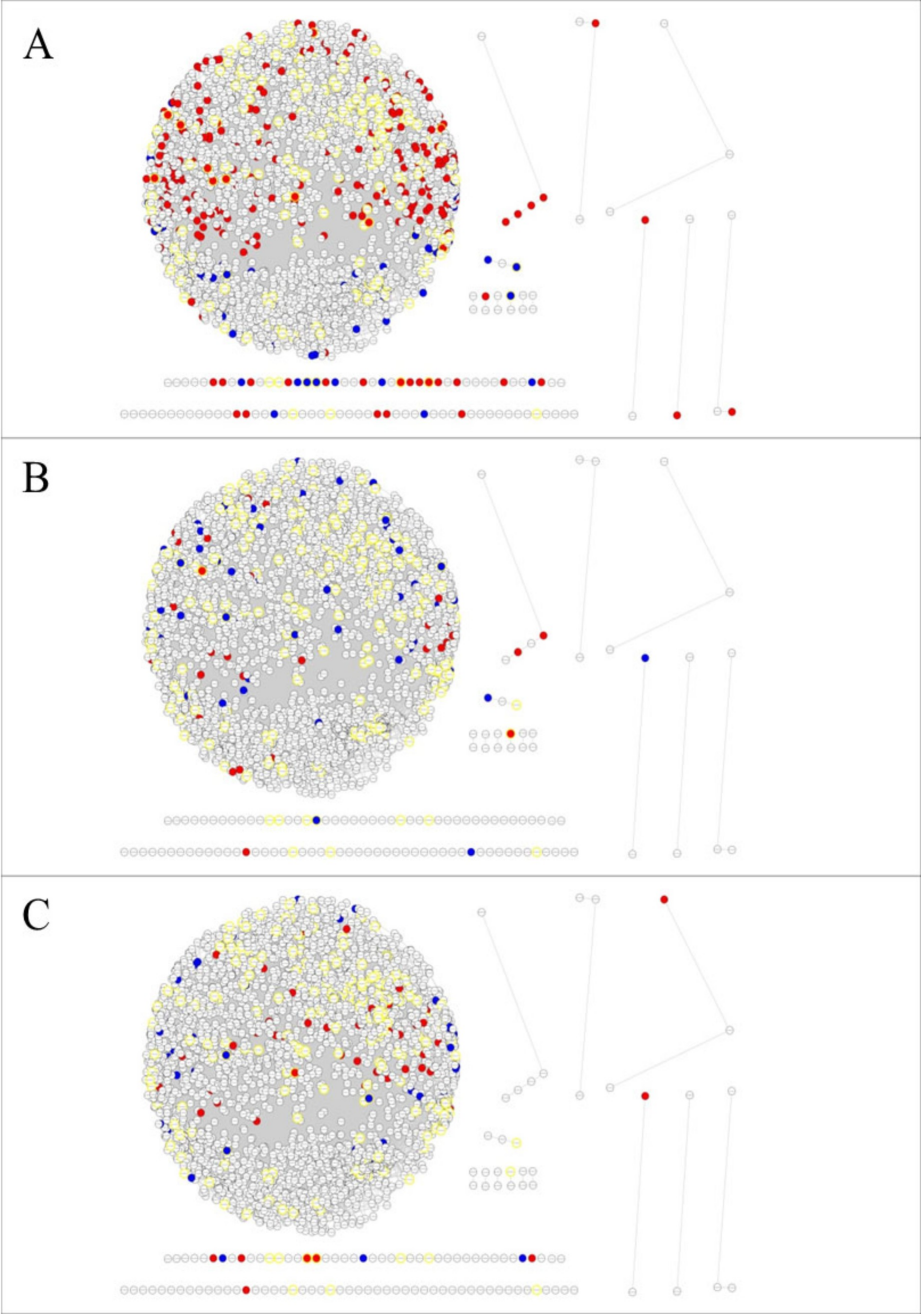
Co-expression networks by aluminium stress. Gene co-expression networks between genes positively (blue) and negatively (red) regulated by aluminium stress at 6 h (A), 54 h (B) and 246 h (C). Circles with yellow borders refer to the genes annotated as transcription factors using the *TF prediction* software from the *PlantTFDB* database.

To elucidate the relationship between genes with two-fold up or downregulation at different exposure times and the other differentially expressed genes, co-expression analysis was performed. For this purpose, the expression values of differentially expressed genes in at least one library were used to construct a gene co-expression network using Spearman's correlation. From the total of 3272 differentially expressed genes, only 92 genes had no connections with *ρ*≥|0.85|, and the others 3180 formed 34,988 connections among them (Fig 1).

Approximately 56% of the genes were directly connected to the two-fold stress-regulated genes at 6 h, 20.6% to the two-fold regulated genes at 54 h and 28.3% to 246 h group of two-fold regulated genes. The 344 genes that were two-fold up or downregulated had only 84 connections among them; however, these genes were directly connected to another 2306 genes by 32,316 connections, revealing the complexity of the plant response to the presence of Al.

Although there were more genes related to those that were two-fold regulated under early stress conditions, this group (G6h) had the lowest mean number of connections, 26 connections per gene, followed by the 54 h group (G54h) with 35.06 connections per gene and the 246 h group (G246h) with 86.40 connections per gene. The high number of connections in G246h reflects the high correlation between their expression profiles in the applied treatments, which indicates the similar transcriptional regulation of these genes in response to stress.

Most genes in this co-expression network had few connections. However, key genes or hubs, consisting of those involved in several stages and pathways of stress, tend to have a greater number of connections. Thus, the identification of these genes is important in determining which are the main genes related to the stress. The analysis of the ten genes with more connections in the general network (hubs) revealed that nine of them were within G246h, corroborating with the high mean number of connections per gene on this group, as none of the hubs appears in the G6h. The gene with the highest number of connections in the general network (main hub) is within G246h and encodes a protein similar to ALS3 from *Arabidopsis thaliana*, which is an ABC transporter associated with aluminium tolerance mechanisms, that affect the redistribution of Al accumulated in sensitive tissues [29,30].

Interestingly, another hub in G246h belongs to the *Multidrug and toxic compound extrusion* (*MATE*) family, similar to proteins such as EcMATE1 from *Eucalyptus camaldulensis*, that performs citrate efflux in the rhizosphere in response to aluminium stress [31] and AtFRD3 from *A*. *thaliana* that mediates the transport of iron complexed with citrate in the xylem [32]. In addition to such transporters, the G246h group has two genes encoding proteins of the C2H2 transcription factor family, similar to STOP2 and STOP1 (*Sensitive to proton rhizotoxicity*) proteins from *A*. *thaliana*, which are the main regulators of aluminium tolerance response in this species and specifically activate the transcription of *MATE* genes and the *ALS3* gene [33,34].

The transcriptional regulation performed by the transcription factors is a key aspect of a stress response understanding, as they are responsible for triggering the transcription of several other stress-related genes and, consequently, this knowledge has a great application potential in plant breeding [35]. In view of this rationale, a total of 203 genes were identified from probes differentially expressed in the analysed libraries (S3 Table), which were annotated as transcription factors using the *TF prediction* software from the *PlantTFDB* database [36]. A variation of two-fold in gene expression was identified in only 25 genes as a result of aluminium treatment (Table 2) and these ones were considered for further exploration. All 25 stress-related transcription factors were regulated as early as at 6 h of exposure to aluminium, and only eight and six of them were still regulated by the stress at 54 and 246 h, respectively.

**Table 2:** Transcription factors related to aluminium stress.

P. trichocarpa	Annotation	6h Regulation	54h Regulation	246h Regulation
**Potri.017G099800.1**	MIKC-MADS	Negative	Positive	Negative
**Potri.019G011500.1**	GRAS	Positive		Positive
**Potri.015G016100.1**	B3	Positive	Positive	Negative
**Potri.015G017000.1**	B3	Positive		Negative
**Potri.001G137800.2**	HD-ZIP	Negative	Positive	
**Potri.012G133800.1**	ARR-B	Negative		
**Potri.003G103500.1**	NAC	Negative		
**Potri.009G117200.2**	MYB_related	Negative		
**Potri.014G047000.1**	ERF	Negative		
**Potri.010G143600.1**	GRAS	Negative		
**Potri.001G214900.1**	M-type-MADS	Positive		
**Potri.001G220500.1**	NAC	Negative		
**Potri.T050600.1**	ERF	Positive		
**Potri.T144800.1**	MYB	Negative	Positive	Positive
**Potri.002G129900.1**	B3	Negative		
**Potri.001G197000.1**	MYB	Negative		
**Potri.001G461000.1**	bHLH	Negative	Negative	
**Potri.003G214000.1**	C2H2	Negative		
**Potri.012G024200.1**	NAC	Negative		Positive
**Potri.012G133700.1**	LBD	Negative	Positive	
**Potri.005G095100.1**	GRAS	Positive		
**Potri.002G228700.1**	MYB	Negative	Positive	
**Potri.006G048700.2**	MIKC-MADS	Negative		
**Potri.006G105300.1**	WRKY	Positive	Negative	
**Potri.010G167500.1**	MYB	Negative		

Genes annotated as transcription factors with variation in gene expression higher than two-fold due to the effect of the aluminium treatment.

Among the transcription factors regulated at 54 and/or 246 h, only *Potri*.*019G011500*.*1* (*GRAS*) and *Potri*.*001G461000*.*1* (*bHLH*) had the regulation similar to that observed at 6 h. This finding demonstrates that the plant's response to stress occurs in the first few hours of exposure' with increased expression of transcription factors, and as the plant remains under stress, the expression variation of these transcription factors tends to decrease.

Transcription factors of the *bHLH* family are widely distributed in several kingdoms, with members characterized in humans, mice, mosquitoes, yeasts and various plants [37], but there is still no member characterized in plants that is directly related aluminium toxicity stress response. However, some members of this family in plants, such as *Root hair defective six-like 2* and *3* and *4* (*RSL2*, *RSL3*, *RSL4*), are positive regulators of lateral root development [38,39]. The inhibition of root development due to the presence of aluminium could be related to the negative regulation of the identified member of the *bHLH* family. Similarly, the GRAS family of transcription factors has a member in plants (*Scarecrow-like 28*, *SCL8*) that regulates the elongation and division of the root cells [40], the main organ affected by the aluminium stress.

**Fig 2:**
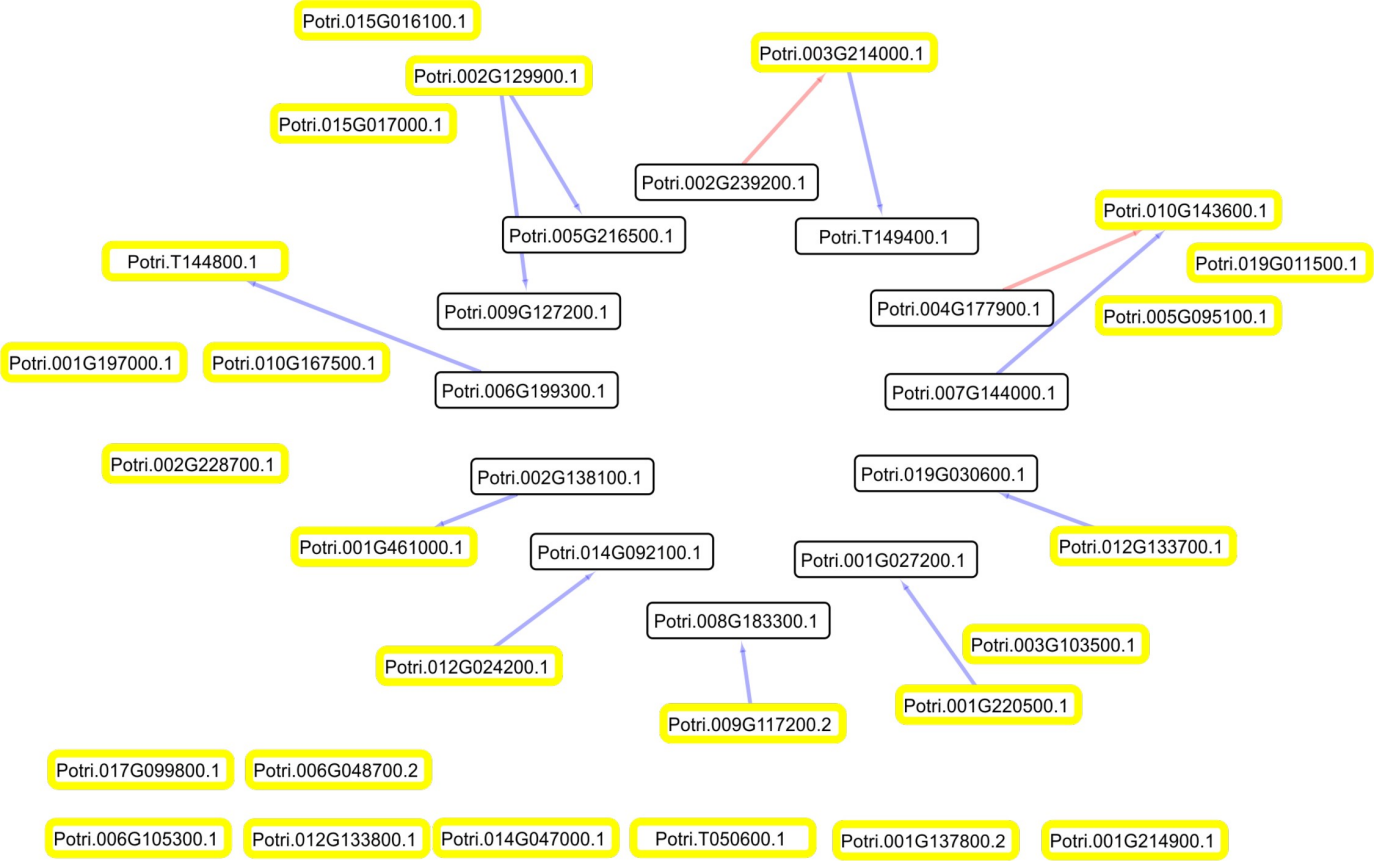
Co-expression network between aluminium-regulated transcription factors. Co-expression network between transcription factors with transcriptional variation greater than 100% due to effect of stress and genes correlated with the same level of variation. Network constructed with correlations with *ρ≥|0*.*8|*. Circles with yellow borders refer to transcription factors, blue lines refer to positive correlations and red lines to negative correlations.

Among these aluminium-regulated transcription factors, 18 are negatively regulated by the stress at 6 h, and these regulators have few connections with other genes that had transcriptional variation two-fold up or down due to aluminium stress (Fig 2). Among the genes discussed above, *Potri*.*019G011500*.*1* had no connections and *Potri*.*001G461000*.*1* was connected only with a single protein with undefined domain.

Another transcription factor of the *GRAS* family (*Potri*.*01G143600*.*1*) that is negatively regulated by the stress at 6 h was connected with genes that have protein kinase and phosphatase domains. In addition, a transcription factor from the *LBD* family (*Potri*.*012G133700*) and another from the *AP2/B3* family (*Potri*.*002G129900*), also negatively regulated, were correlated with genes with protein kinase and auxin-responsive domains. Interestingly, the gene with the auxin-responsive domain (*Potri*.*009G127200*) belongs to the *SAUR* (*Small auxin upregulated*) family, and it has previously been shown that the expansion of hypocotyl cells in *A*. *thaliana* is influenced by the action of genes from this family along with protein kinases and phosphatases [41]. It is possible that the negative regulation of these connected genes is associated with inhibition of root cell expansion due to aluminium toxicity.

**Fig 3:**
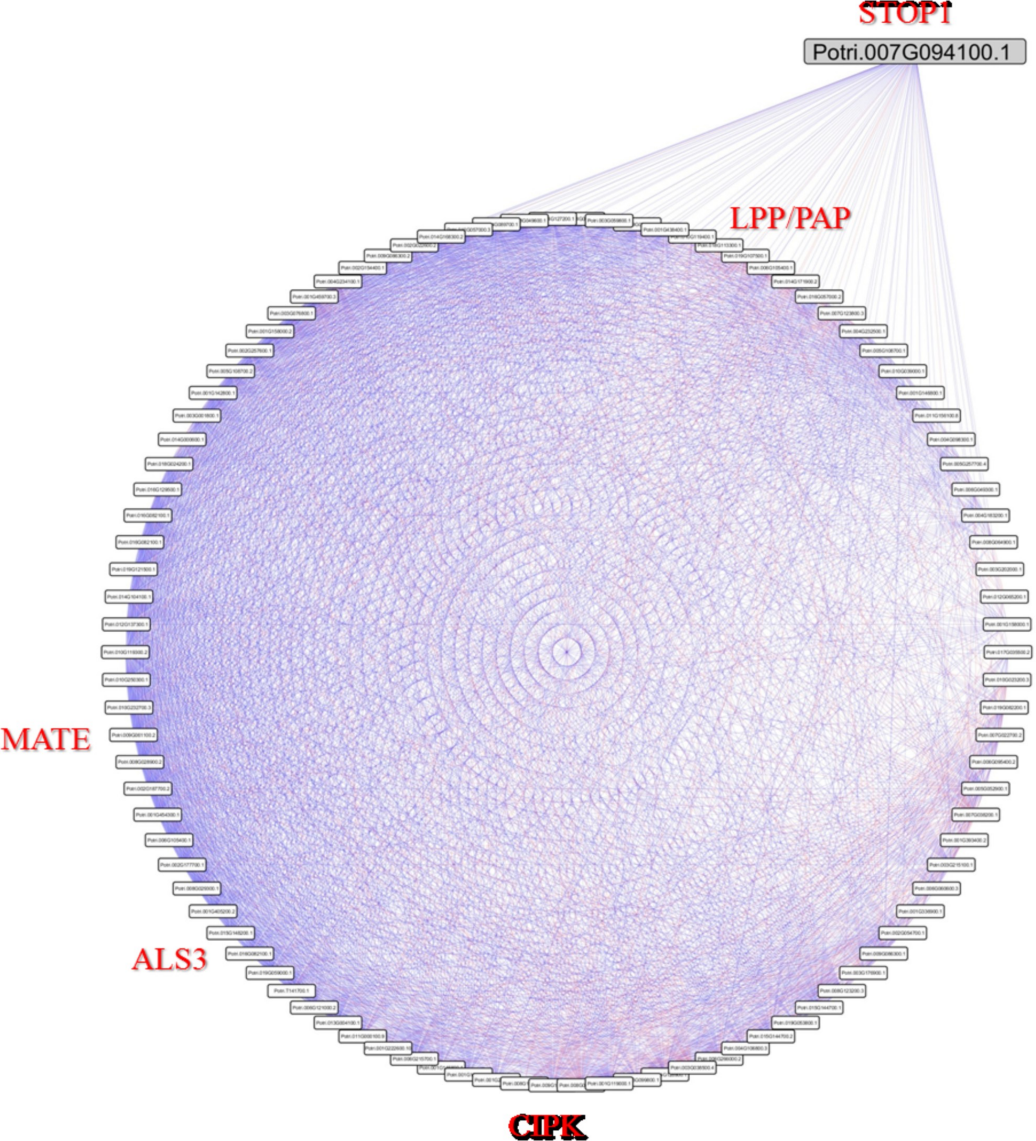
Network of *Potri*.*007G094100*.*1*. Direct connections of the *Potri*.*007G094100*.*1* gene, annotated as transcription factors of the *C2H2-zinc finger* family, orthologous to *AtSTOP1* from *A*. *thaliana* and the main hub between the transcription factors identified. Blue lines refer to positive correlations and red lines to negative correlations between the *Potri*.*007G094100*.*1* gene and the other 100 genes to which it is correlated, with *ρ≥|0*.*8|*.

The 203 transcription factors (total) were connected to another 1495 genes by 26,343 connections, while the 25 that are two-fold regulated by the stress are connected to only 12 genes by 12 connections. *Potri*.*007G094100*.*1* is the most connected gene (with 100 connections to other genes) within the group annotated as transcription factors and was classified as belonging to the family *C2H2-zinc finger* (Fig 3). This family comprises genes previously characterized in several species as involved in the regulation of the aluminium stress response, where the *AtSTOP1* gene and its homologues in the other species studied are the main regulators of the Al tolerance mechanism [33,42,43].

The analysis using the *blastp* algorithm against the *A*. *thaliana* proteome revealed that the gene most similar to *Potri*.*007G094100*.*1* is *At1G34370*, the already well characterized *AtSTOP1*. Therefore, this support the possibility that *Potri*.*007G094100* is responsible for regulating Al response mechanisms in *Populus*. The regulation of aluminium tolerance, as discussed above, is triggered by the sensitivity of the root cells to Al^3+^ and H^+^, resulting in the regulation, usually post-translational, of the STOP1 protein, which in turn, activates genes related mainly to the exudation of organic acids (such as *MATEs* and *ALMTs*), ion homeostasis (such as *SULTR3*, *NRAMP3* and *HAK5*) and aluminium translocation (such as *ALS3*) [33,43]. Normally, the regulation of the *STOP1* gene is correlated with that of genes encoding protein kinases, an observation likely related to the differential regulation of *STOP1* in response to changes in Al^3+^ and H^+^ [43].

Among the 100 genes with transcriptional profile correlated with *Potri*.*007G094100*.*1*, four genes have sequence homology with already identified genes that are regulated by AtSTOP1 in *A*. *thaliana* [33], including *Potri*.*009G061100*.*2*, *Potri*.*016G082100*.*1*, *Potri*.*001G222600*.*1* and *Potri010G119400*.*1*, which are similar to the *AtMATE*, *AtALS3*, *AtCIPK23*, and *AtLPP3*/*AtPAP1* genes, respectively. The first two genes, *AtMATE* and *AtALS3*, as noted above, are involved with the citrate efflux and aluminium transport, respectively, whereas *AtCIPK23* is related to the regulation of ion transport and *AtLPP3*/*AtPAP1* are phosphatidic acid phosphatases [33].

In addition to these previously characterized genes similar to *AtSTOP1* transcription factor targets in *A*. *thaliana*, it was possible to identify conserved domains in the other proteins encoded by the genes correlated with *Potri*.*007G094100*.*1*, which may suggest involvement with responses to aluminium toxicity. Such proteins include those with glycosyl-transferase domains, because proteins of this type have been shown to be activated by aluminium, associated with cell wall modifications and protection against reactive oxygen species [44], and Serine/threonine kinases, which have already been associated with the development of roots under phosphate deficiency stress in acid soils [45]. Four genes with the *Glycosyl transferase* domain and three with the *Serine/threonine kinase* domain were identified.

**Fig 4:**
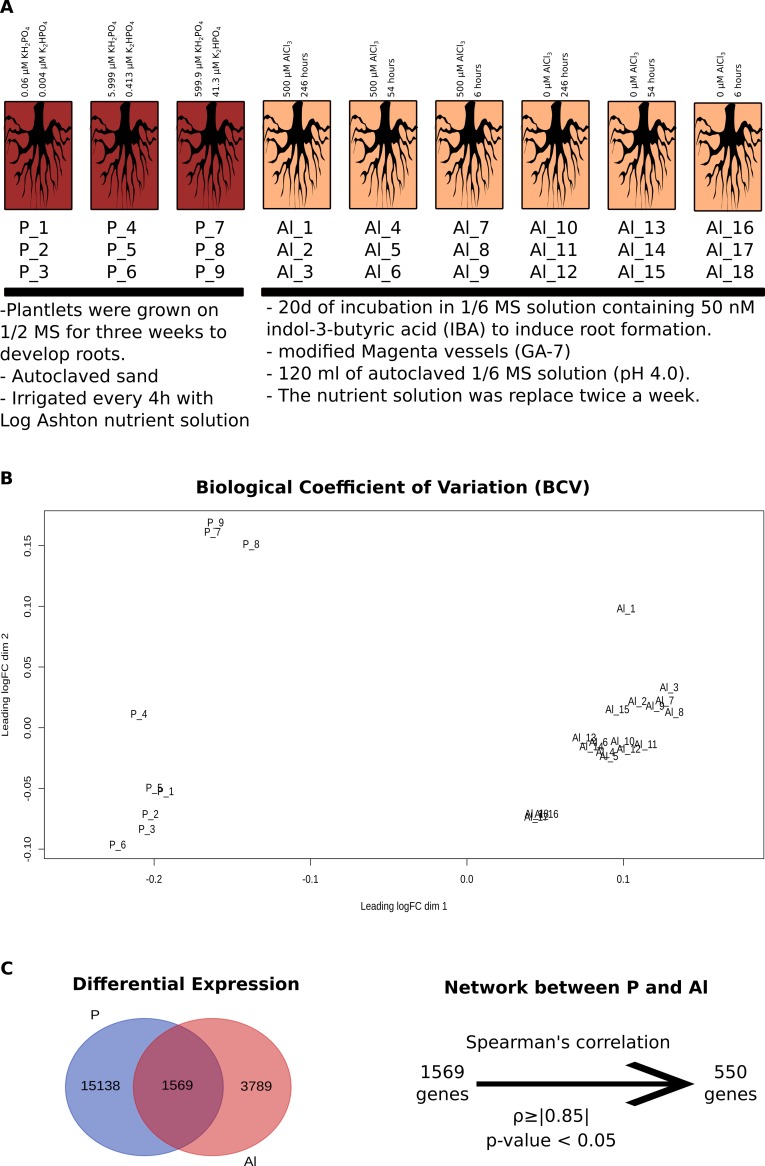
Description of phosphorus and aluminium libraries. A—Description of the *Populus* plants cultivation conditions and the specificities of the stresses; B—Quantification of similarity between libraries based on two dimensions taking into account the biological variability coefficient (BVC) of each library; C—Description of the filters used to select the genes for the construction of the P and Al network.

In addition to the high concentration of toxic aluminium in acid soils, the low availability of free phosphate is limiting for agricultural practices [1]. Some responses to phosphate deficiency are similar to responses to aluminium toxicity, such as the exudation of organic acids that can chelate toxic Al^3+^ ions and make available the phosphate that was bound to the metal [46]. Therefore, identifying common responses within the two stresses is an interesting strategic approach in order to improve the adaptation of crops to this environment. This approach is possible as, in these acid soils, plants face both stresses simultaneously, possibly leading to some co-opted molecular responses that can be explored. To compare the transcriptional regulation and the relationships between aluminium- and phosphate deficiency-activated genes, microarray data on *Populus* spp. roots under high, medium and low phosphate concentrations were used (Fig 4A and S2 Table). Although P deficiency and Al toxicity are normally associated, it can be observed from the data that they exhibit opposite transcriptional behaviour (Fig 4B). In dim1 there is a clear separation between the P and Al libraries, while in dim2 the Al libraries are clustered and there is a separation between the severe phosphorus deficiency libraries and the other P libraries.

**Fig 5:**
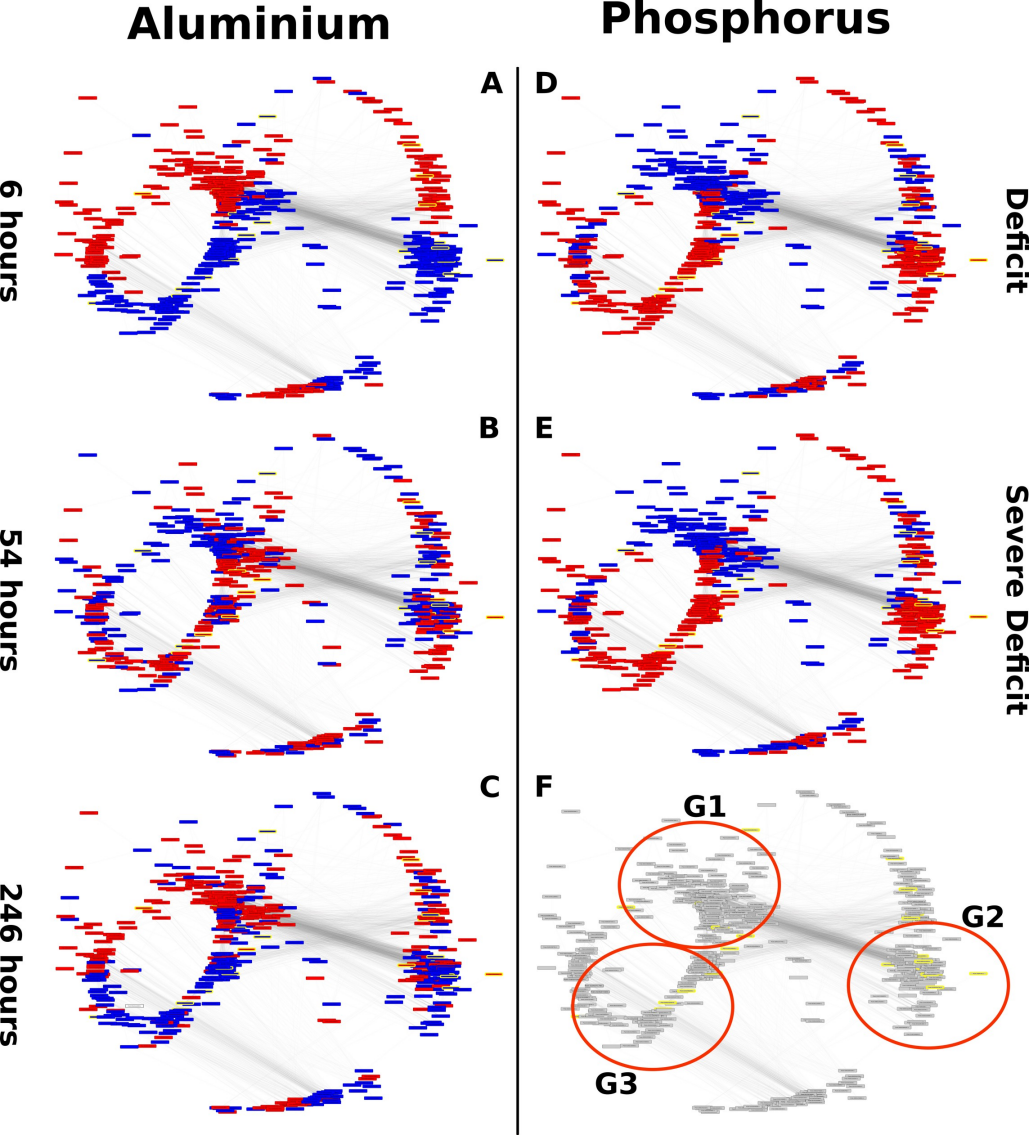
Co-expression networks between genes regulated by phosphate deficiency and aluminium toxicity. The green colour represents genes regulated positively (stress/normal condition > 1) and the red colours genes regulated negatively (stress/normal condition < 1). A- Regulation by aluminium stress at 6 h; B- Regulation by aluminium stress at 54 h; C- Regulation by aluminium stress at 246 h; D- Regulation by normal phosphate deficiency; E- Regulation by severe phosphate deficiency; F- groups of genes with different regulation between the two stresses, G1, G2 and G3.

A total of 550 differentially expressed genes were identified in both the experiments with phosphate deficiency (9 libraries) and aluminium stress (18 libraries) that have expression profiles with correlation *ρ≥|0*.*8|* between them (Fig 4C). These genes presented 8380 connections, and from the analysis of the transcriptional variation between aluminium stress and phosphate starvation stress, it was possible to identify three major groups differentially regulated by each stress (Fig 5). Differentially expressed genes considered as under mild phosphorus stress were those with significant variation between the samples of plants cultivated on average and high phosphate concentration, whereas the ratio between the samples of plants cultivated on high and low phosphate concentration were used for defining severe stress (low phosphate/high phosphate).

The group definition shown in Fig 5 was based on the topology of the co-expression network between genes regulated by phosphate deficiency and Al toxicity. Group 1 (G1) is composed of genes (1) regulated positively at 6 and 246 h of exposure to aluminium stress, (2) regulated positively and negatively at 54 h of exposure to the same stress and (3) regulated negatively by normal and severe phosphate deficits. Groups 2 and 3 (G2 and G3) show the opposite profile to that reported for G1, and G2 genes are the most distant from the other groups. Given that aluminium stress caused more transcriptional, physiological and morphological changes at 6 h [47], it can be inferred that genes in G1 are mostly activated by toxic aluminium and inhibited during the phosphate deficit, whereas the genes allocated in G2 and G3 have the opposite profile, being downregulated by aluminium and upregulated by the phosphate deficit.

To analyse the specificities of each group, a *Blast2GO* analysis was performed for G1, G2 and G3 separately (S3 Table). Regarding the "*Cellular component*" class, G1 and G2 presented a similar distribution profile among the subclasses "*Membrane*", *"Intracellular"* and "*Organelle*", with a larger number of sequences in the second subclass ("*Intracellular"*), followed by "*Membrane"* and finally "*Organelle"*. For the G3 genes, *"Organelle"* was the second most representative subclass (Table 3). In addition, it is interesting to note that G1 has genes in the *"Ribosome"* subclass (11 genes), which is exclusive to this group; G2 comprises genes possibly related to photosynthesis, as the subclasses *"Chloroplast"* (3 *genes)*, *"Photosynthetic membrane"* (3 genes) and *"Thylakoid part"* (3 genes) are exclusively present in this group; and G3 has the largest number of genes allocated in the subclasses *"Nucleus"* (7 genes) and *"Cytoskeleton"* (4 genes).

**Table 3:** Discrimination of distribution profiles of genes belonging to groups G1, G2 and G3 in classes and subclasses of the first level of analysis in the Blast2GO software.

Subclasses (Blast2GO)	Classes (Blast2GO) and percentages of genes belonging to the total of each group		
**Celular component**			
	**G1 (28,73%)**	**G2 (20,83%)**	**G3 (25%)**
**Membrane**	11,49%	10,41%	7,26%
**Intracellular**	15,51%	14,58%	17,74%
**Organelle**	10,34%	9,37%	12,90%
**Molecular function**			
	**G1 (63,21%)**	**G2 (56,25%)**	**G3 (45,40%)**
**Catalytic activity**	37,93%	15,62%	27,42%
**Binding**	30,45%	40,62%	52,42%
**Biological function**			
	**G1 (51,15%)**	**G2 (37,5%)**	**G3 (47,58%)**
**Metabolic process**	37,36%	27,08%	29,84%
**Cellular process**	33,33%	27,08%	32,25%
**Localization**	10,34%	5,21%	8,87%
**Biological regulation**	6,32%	6,25%	11,29%
**Single organism process**	22,99%	21,87%	12,90%
**Cellular component, organization or biogenesis**	10,34%	6,25%	5,64%
**Response to stimulus**	n.d.	3,12%	4,03%

The percentages refer to the number of genes relative to the total number of genes in each group.

For the "*Molecular function*" class, G2 and G3 have more genes allocated to subclass *"Binding"* than to the "*Catalytic activity*" subclass, as opposed to G1 (Table 3). G1 is the only group that has three subclasses related to catalytic activity—*"Oxidoreductase"* (13 genes), *"Transferase"* (22 genes) and *"Hydrolase"* (19 genes)—whereas G1 has no genes in the subclasses *"Protein binding"* and "*DNA binding*". It is also worth noting that G3 is the only group with genes assigned to the subclasses *"Protein kinase activity"* (eight genes) and *"Pyrophosphate activity"* (eight genes).

For the "*Biological process*" class, there was no similarity between the distribution of the genes of each group in the subclasses; however, G2 and G3 had a more similar profile than G1 (Table 3). With respect to the specificities of each group, G1 was the only group with genes allocated to subclass *"Cellular amino acid metabolic process"* (nine genes), G2 included genes allocated to the subclass *"Photosynthesis"* (four genes), and G3 had genes allocated to various classes related to RNA metabolism, such as *"RNA metabolic process"* (14 genes), *"RNA processing"* (five genes), *"ncRNA metabolic process"* (six genes), and *"Transcription*, *DNA-template"* (six genes).

The genes in G1, which are upregulated by aluminium, presented specific characteristics in the Blast2GO analysis that were consistent with those observed physiologically and morphologically in aluminium-stressed plants. The high number of genes (compared to G2 and G3) annotated with catalytic function, such as transferases, hydrolases and oxidoreductases, regulated positively by aluminium and not by phosphate deficiency may be related to the protection mechanisms activated by the plant to cope with the stress [44,47]. Analyses of G2 genes did not reveal any specificity, although the results revealed a few genes possibly involved in photosynthesis, a finding that is not conclusive because only root libraries were used in this analysis. As noted above, it is possible that some genes in G2, despite being activated by phosphate deficiency, are involved in processes not very distant from those related to the genes in G1 because the criterion used to define gene regulation was restrictive and the genes of these two groups are connected in the co-expression network.

Genes that were upregulated by phosphate but not by aluminium grouped in G3 were allocated in classes related to protein kinases, pyrophosphatases and RNA metabolism-related processes. Considering the distance of this group in the co-expression network (Fig 5) relative to G1 and G2 and the specific profile identified by the Blast2GO analysis, it is interesting to note that a considerable part of the phosphate deficiency response regulation is dependent on post-transcriptional and post-translational modifications [22]; therefore, the genes allocated to G3 may be related to the regulation of the mechanisms of response to phosphate deficiency.

To identify mechanisms common to both stresses, it is necessary to analyse the genes that have the same regulation profile for responses to aluminium toxicity and to phosphate deficiency. The genes transcribed at 6, 54 and 246 h of stress exposure had differences in the type of transcriptional regulation (positive or negative). Therefore, for the purpose of comparison, the regulation data at 6 h of aluminium stress and the data for severe stress due to phosphate deficiency were used, as these were the samples of the two stresses with most transcriptional variation.

**Fig 6:**
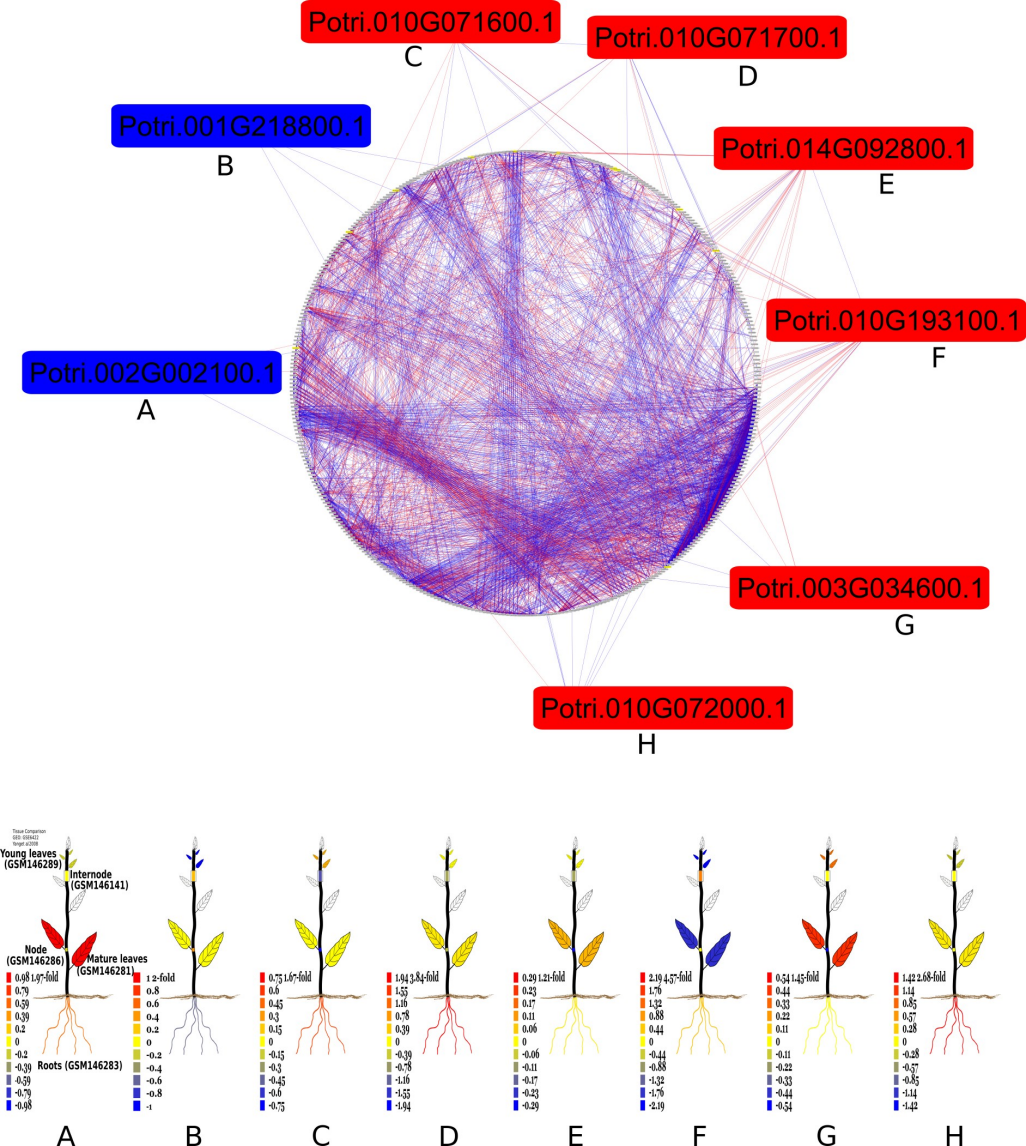
Co-expression network between genes with the same transcriptional regulation profile for aluminium toxicity and phosphate deficiency. Green rectangles refer to the activated genes and red rectangles to genes inhibited by both stresses; grey rectangles at the edge of the circle refer to genes with constant expression between the two stresses (variation below 20%). Blue lines refer to positive correlations and red lines to negative correlations, with *p≥0*.*8*.

In this context, transcriptional variation equal to or greater than 20% was used as the criterion for positive or negative regulation so that genes with similar behaviour within the two stresses could be identified. Genes that were negatively regulated in one stress and positively in another were excluded from the analysis. Finally, a co-expression network was generated with 380 genes connected by 4159 connections (Fig 6).

Of the total, only eight genes have transcriptional variation equal to or greater than 20%, where five are positively regulated and two negatively regulated, by both stresses (Table 4). These eight genes are connected to another 77 genes by 486 connections and, among the regulated genes, *Potri*.*010G193100* is the one with the highest number of connections, with 37 in total. To analyze the possible functions of genes regulated by both stresses, these were annotated with *blastp* against the *A*. *thaliana* database (Table 4) and only one protein, encoded by the *Potri*.*002G002100* gene, presented similarity with proteins of yet unknown function.

**Table 4:** Description of the genes of *P*. *trichocarpa* with the same regulation profile for phosphate deficit and aluminium toxicity.

Gene	*Best hit in A*. *thaliana*	*A*. *thaliana annotation*	Variation		Regulation
			Phosphorus	Aluminium	
**Potri.003G034600**	AT1G17710.1	*AtPEPC1* (fosfoetanolamina/fosfocolina phosphatase 1)	1,845046	1,220214	**positive**
**Potri.010G071600**	AT2G38940.1	*AtPT2/AtPHT1;4* (phosphate transporter)	1,673688	1,230394	**positive**
**Potri.010G071700**	AT2G38940.1	*AtPT2/AtPHT1;4* (phosphate transporter)	1,561246	1,226014	**positive**
**Potri.001G218800**	AT3G44350.2	*AtANAC61* (contains NAC domain)	0,745537	0,753407	**negative**
**Potri.002G002100**	AT1G21010.1	*unkown*	0,748707	0,631502	**negative**
**Potri.010G072000**	AT2G38940.1	*AtPT2/AtPHT1;4* (phosphate transporter	1,738861	1,274632	**positive**
**Potri.014G092800**	AT5G24090.1	*AtCHIA/AtLYS1* (kitinase A/Lisozima1)	1,254137	2,475638	**positive**
**Potri.010G193100**	AT2G38080.1	*AtALMCO4* (Laccase-like multi-copper oxidase)	1,226961	4,14973	**positive**

Half of the genes identified in this analysis, *Potri*.*003G034600*, *Potri*.*010G071600*, *Potri*.*010G071700* and *Potri*.*010G072000*, were related to functions already reported as induced by phosphate deficiency. The first gene is similar to *AtPEPC1*, which is reported to be responsive to phosphate deficiency, and the protein encoded by it is related to lipid hydrolysis (phosphoethanolamine) for mineralization and internal recycling of phosphate under deficit conditions [23]. The last three genes mentioned are similar to *AtPT2/AtPHT1;4*, which is one of the major genes encoding phosphate transport proteins and mediate the influx of this element from the rhizosphere into the root [24]. In addition, these three genes have already been studied in *Populus* spp. and are annotated as *PtPHT1*.*5* (*Potri*.*010G071700*), *PtPHT1*.*6* (*Potri*.*010G071600*) and *PtPHT1*.*7* (*Potri*.*010G072000*), members of the phosphate transporter family. *PtPHT1*.*6* is exclusively expressed in roots and, together with *PtPHT1*.*5*, is more strongly expressed under normal and low phosphate conditions than under high phosphate conditions [48].

These four genes identified in *Populus* are activated in the two stresses with variation greater than 20%. It is possible that positive regulation of the gene *Potri*.*003G034600 (PEPC)* in the presence of aluminium is associated with changes in the phospholipid layer of the root cell membrane, because this is the first site of interaction with the Al^3+^ ion, and that the activation of phosphate transporters is related to the reduction of the availability of this element caused by the aluminium stress condition, suggesting the possibility of co-opted response strategies to these two stresses. Regarding the putative phosphate transporters, it was already reported that these can be upregulated by aluminium stress in ryegrass (*Lolium perene*), even without phosphate starvation stimuli [49] and, this observation together with the reported here, raises the question whether the transcriptional variation of these genes related to phosphate starvation is a direct response that helps the plant to cope with aluminium stress or are part of an evolutionary co-opted adaptation to acid soils, where the both stresses co-exists.

The gene *Potri*.*001G218800* encodes a protein similar to AtNAC61, which was not specifically characterized, but in *Oryza sativa*, approximately 25 transcription factors members of the NAC family are regulated by exposure to toxic aluminium, and most are upregulated by the stress [50]. In the case of the present analysis, this putative transcription factor was inhibited by both aluminium stress and phosphate deficiency, an effect not previously described in the literature. However, this family of transcription factors is broad and associated with varied responses to different stresses [51], and therefore, more studies are needed to associate the roles of these genes in the stresses analysed here.

Likewise, the literature has yet no report on relationships involving the *AtCHIA/AtLYS1* gene from *A*. *thaliana* (similar to *Potri*.*014G092800*), but this gene encodes a protein with lysozyme activity, and it is possible that this high-molecular-weight carbohydrate breakdown activity is associated with the modifications that can occur in the plasma membrane due to both stresses studied. Finally, the gene *Potri*.*010G0193100* encodes a protein similar to that encoded by *AtALMCO4/LAC4* that is related to lignin biosynthesis [52]. Interestingly, there are reports of lignin deposition on roots of *Triticum aestivum* under aluminium stress [53] but not for phosphate deficiency.

**Fig 7:**
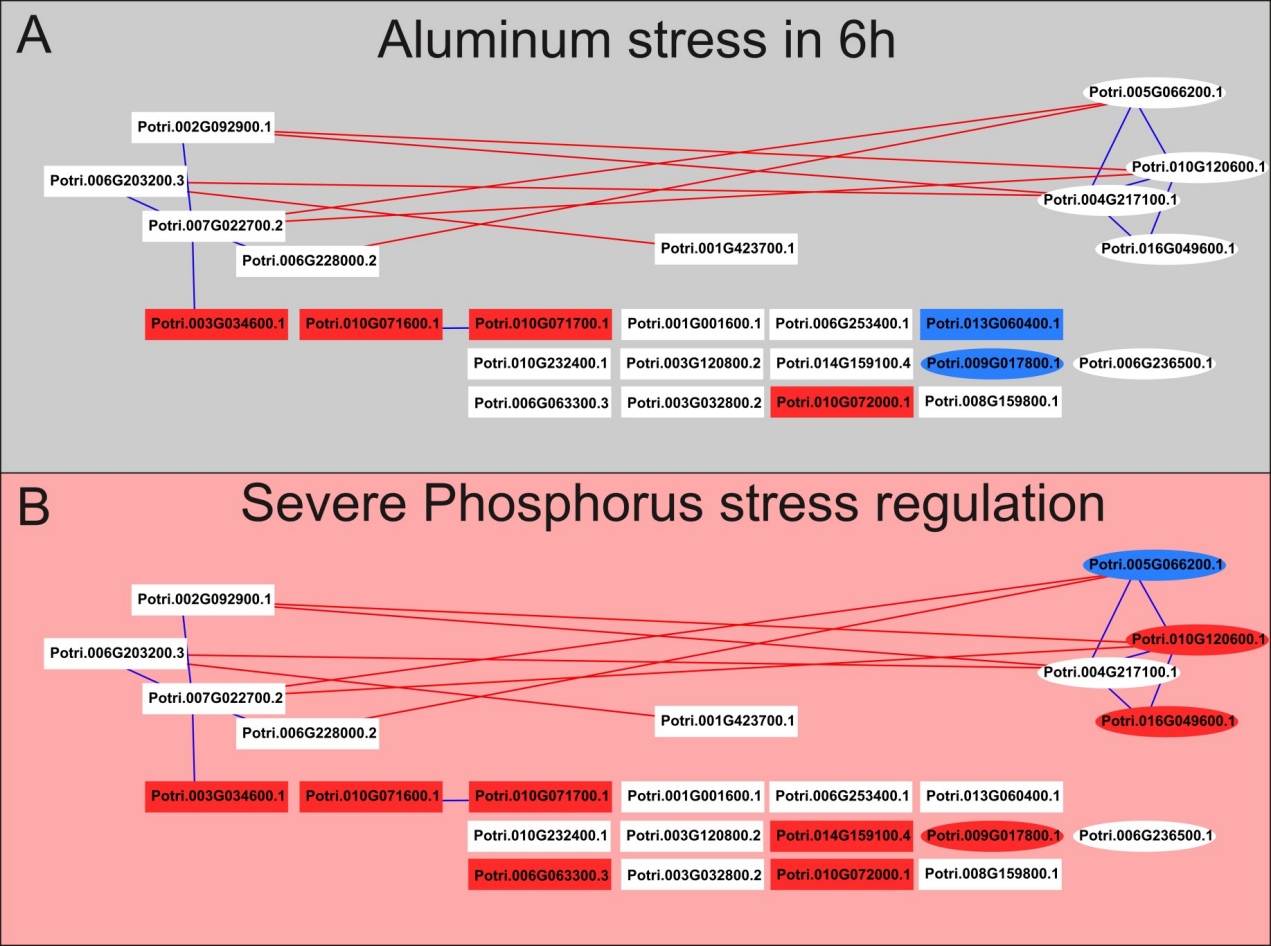
Correlations (*ρ*≥|0.8|) between genes encoding proteins similar to those responsive to phosphate deficiency (rectangles) and regulated by the transcription factor *STOP1* from *A*. *thaliana* (ellipses). A- Regulation profile at 6 h of aluminium stress; B- regulation profile under severe phosphate deficiency stress. Green represents positive regulation, and red represents negative regulation; blue lines represent positive correlations, and red lines represent negative correlations.

In an analysis of data from four genome-wide transcriptome experiments with *A*. *thaliana* subjected to phosphate starvation, 95 responsive genes to this stress were identified in all experiments and were named as *"core PSR genes"* (core phosphate starvation response genes) [22]. For the toxic aluminium stress, as previously discussed, the known regulation network is associated with genes regulated by the *STOP1* transcription factor in *A*. *thaliana* [33]. Therefore, a *blastp* analysis was performed to enable the identification of genes encoding proteins similar to those regulated by *STOP1* in *A*. *thaliana* and proteins encoded by the "*Core PSR*" genes. Only genes from the co-expression network related to aluminium stress and phosphate deficiency were used in this analysis, in order to observe the relationships within them (Fig 7).

As can be observed, genes that are similar to those regulated by *STOP1* in *A*. *thaliana* do not have positive correlations with the "*Core PSR*" genes. This suggests that although the two stresses occur simultaneously in acid soils, there may be specific response mechanisms associated with each stress. These genes have been annotated with several functions already related to phosphate starvation and response to aluminium toxicity (S1 and S3 Tables)[22,33] and may therefore be related to the response to these two stresses in *Populus* spp.

It is worth highlighting that genes similar to those commonly activated by phosphate deficiency did not have direct positive correlations with genes similar to those activated by the transcription factor *STOP1* in *A*. *thaliana*, a key regulator of aluminium tolerance, which shows the differences between the main responses to these two stresses observed in acid soils.

In addition, eight genes with the same regulation profile in response to both stresses were identified in the present study, suggesting that these genes may be promising targets of future studies for better understanding the interconnected tolerance mechanisms for phosphate deficiency and aluminium toxicity. The analyses of transcriptional modifications related to phosphate deficiency and aluminium toxicity stresses by the construction of gene co-expression networks revealed specific characteristics of the responses to each stress as well as some molecular features common to both, which should be analysed more deeply and confirmed by further experiments. The data generated in this work can support further studies regarding the association of genes to important aspects of the adaptation of woody crops to acid soils,

## Materials and methods

### Analysis of data from microarray experiments

The microarray data used in this work were obtained from accession numbers GSE19297 and E-MTAB-3934. Regarding the first dataset, Populus roots were treated with Al (500 μM AlCl3) in 1/6 MS medium, replaced twice a week, in modified Magenta vessels (GA-7), for 6 h, 54 h, 246 h, for each time point. Root tips of three independent plants were analyzed, 18 libraries in total. For the second dataset, Populus plantlets were grown into PVC tubes filled with autoclaved sand and grown in a greenhouse, the plants were automatically irrigated every 4 hours with Long Ashton nutrient solution containing high phosphate supply (599.9 μM KH_2_PO_4_, 41.3 μM K_2_HPO_4_), mildly Pi starved (5.999 μM KH_2_PO_4_ and 0.413 μM K_2_HPO_4_) and Pi starved (0.060 μM KH_2_PO_4_ and 0.004 μM K_2_HPO_4_), six plants per treatment were used and the roots, fine roots (<2 mm diameter), of two individual poplars were pooled yielding three biological replicates per treatment, totalling nine libraries.

Microarray analyses were carried out with the Affymetrix GeneChip poplar genome array (v1.1 from the *P*. *trichocarpa* genome project), data were analysed using the free statistical software R (R version 3.3.2) following the protocol described by Janz [54]. The R package "*affy*"[55] was used to normalize the probes using the "*rma*" function, available in the Bioconductor software [56]. The log_2_ expression values of the transcripts that were present in all replicates, obtained by the "*mas5calls*" function in at least one of the conditions, were used for the subsequent analyses. Student’s t-test (*p*<0.05) was used to identify the genes that were differentially expressed in treatments with aluminium as well in doses of phosphate. The genes considered as differentially expressed were those genes that showed (1) expression in all replicates in at least one library and (2) abundance of the transcript in at least one treatment significantly different from that in another treatment according to Student's t-test, that is, differential expression in at least one library.

For annotation of differentially expressed transcripts according to the *Populus trichocarpa* genome, the *Blastx* tool was used to align the probes with the CDS database of *P*. *trichocarpa*. An e-value < 1e-5 and a maximum of 10 alignments was used for each probe, and the best alignment was selected.

### Construction of gene co-expression networks

Two different co-expression networks were prepared from the expression values obtained for the libraries related to each experiment. To identify the proximity of the gene expression profile in the different treatments, the biological coefficient of variability (BCV) was determined for each treatment and an MDS plot was constructed using the EdgeR package. To verify the correlation of the genes expression profile, two analyses were performed, a Spearman’s correlation to identify the relationship between genes and the p-value of this number to determine if this correlation was statistically significant. The p-value was determine using the formula in the Excel *P* = IDIST{ABS[*r*/SQRT({1-r*r}/{*n*-2})],[*n*-2],2} as describe by Usadel [57], where the number of samples is indicated by *n*, and *r* is the observed co-expression score, only correlations with p-value <0.05 were used. Spearman’s correlation with ρ≥0.8 was selected because unlike the value used in the literature, ρ≥0.7, the goal was to focus only in the genes that had the strongest correlation. The first network was based in only differentially expressed genes among the 18 libraries related to the aluminium stress experiment. For the second co-expression network, all genes that were differentially expressed in libraries related to aluminium stress were used together with the libraries with the dose variations of phosphate.

### Enrichment of gene co-expression networks

To establish the type of regulation in relation to stress for each dataset, the following procedures were used: (1) for the aluminium stress-related libraries, the mean values of the biological triplicates of each gene in the different libraries were estimated, and subsequently, the values of the aluminium stress-related libraries were divided by the values of the library for the same stress exposure time but in the control condition; (2) for the phosphate-related libraries, again, the mean expression values for each gene among the biological triplicates of all libraries were estimated, and then the values of the average phosphate concentration library were divided by the values of the high phosphate concentration library (to characterize the stress), and likewise, the low phosphate concentration values were divided by the high phosphate concentration values (severe stress). It is worth noting that the plants in this experiment under average phosphate concentrations showed signs of stress, albeit less severe than those exhibited by the plants under lower concentrations of this element [58].

Using the values obtained in the calculations described above, it was possible to generate the filters used in the work: positive regulation (coefficient > 1), negative regulation (coefficient < 1), transcriptional variation higher than 20% (0.83 ≤ coefficient ≤ 1.2) and transcriptional variation two-fold up or down (0.5 ≤ coefficient ≤ 2.0). For each gene co-expression network constructed, the *P*. *trichocarpa* proteins corresponding to the probes used in the respective network were analysed in the *TF prediction server* software of the *PlantTFDB* database for predicting possible transcription factors present in the network and the family of transcription factors to which it belongs. In addition, the *Blast2GO* software [59] was used to identify enriched *Gene Ontology* classes in the data for the distinct groups of gene co-expression networks.

For the identification of genes similar to those regulated by *AtSTOP1*, the main regulator of transcriptional response to Al toxicity in *A*. *thaliana*, and to those commonly activated by phosphate deficiency (*Core PSR genes*), information from the literature [22,33] was used to identify *A*. *thaliana* proteins and to extract them from the *TAIR* database. With these sequences, a *blastp* analysis was performed against the *P*. *trichocarpa* proteome to identify the proteins potentially regulated by *STOP1* and belonging to the *Core PSR* in this species as well as to screen for the presence of the coding genes in the co-expression networks.

## Supporting information

S1 Table Expression values and annotation of the genes in the co-expression network of aluminium stress. (XLSX)

S2 Table Expression values and annotation of the genes identified as differentially expressed among the phosphate dosage libraries. (XLSX)

S3 Table Gene Ontology (GO) enrichment analysis, transcription factor annotation and *core PSR*/*STOP1* regulated genes annotation. (XLSX)
